# Combined subsegmentectomy: postoperative pulmonary function compared to multiple segmental resection

**DOI:** 10.1186/1749-8090-6-17

**Published:** 2011-02-20

**Authors:** Kentaro Yoshimoto, Hiroaki Nomori, Takeshi Mori, Yasuomi Ohba, Kenji Shiraishi, Koei Ikeda

**Affiliations:** 1 Departments of Thoracic Surgery, Faculty of Life Sciences, Kumamoto University, 1-1-1 Honjo, Kumamoto 860-8556, Japan; 2 Division of General Thoracic Surgery, Department of Surgery, School of Medicine, Keio University, Tokyo, Japan

## Abstract

**Background:**

For small peripheral c-T1N0M0 non-small cell lung cancers involving multiple segments, we have conducted a resection of subsegments belonging to different segments, i.e. combined subsegmentectomy (CSS), to avoid resection of multiple segments or lobectomy. Tumor size, location of tumor, and forced expiratory volume in 1 second (FEV_1_) of each preserved lobe were compared among the CSS, resection of single segment, and that of multiple segments.

**Methods:**

FEV_1 _of each preserved lobe were examined in 17 patients who underwent CSS, 56 who underwent resection of single segment, and 41 who underwent resection of multiple segments, by measuring pulmonary function and lung-perfusion single-photon-emission computed tomography and computed tomography before and after surgery.

**Results:**

Tumor size in the CSS was significantly smaller than that in the resection of multiple segments (1.4 ± 0.5 vs. 2.0 ± 0.8 cm, p = 0.002). Tumors in the CSS were located in the right upper lobe more frequently than those in the resection of multiple segments (53% vs. 5%, p < 0.001). Postoperative of FEV_1 _of each lobe after the CSS was higher than that after the resection of multiple segments (0.3 ± 0.2 vs. 0.2 ± 0.2 l, p = 0.07). Mean FEV_1 _of each preserved lobe per subsegment after CSS was significantly higher than that after resection of multiple segments (0.05 ± 0.03 vs. 0.03 ± 0.02 l, p = 0.02). There was no significant difference of these factors between the CSS and resection of single segment.

**Conclusions:**

The CSS is effective for preserving pulmonary function of each lobe, especially for small sized lung cancer involving multiple segments in the right upper lobe, which has fewer segments than other lobes.

## Background

**Figure 1:**
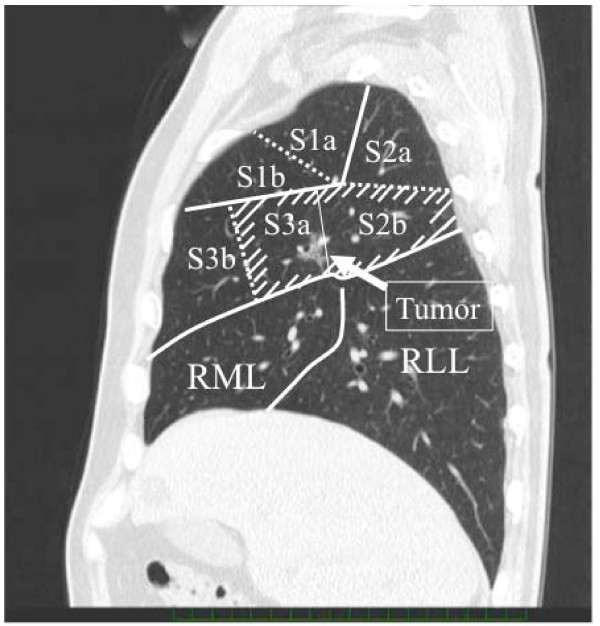
**Sagittal image of CT**. The tumor located between the right subsegment 2b and 3a.

Advances in high-resolution CT scanning have led to frequent detection of peripheral T1N0M0 non-small cell lung cancers (NSCLCs). Several studies have demonstrated the effectiveness of segmentectomy, regarding not only preservation of pulmonary function but also prognosis [1-4]. However, for small peripheral c-T1N0M0 NSCLCs involving multiple segments, resection of entire segments damages pulmonary function to the same extent as lobectomy. To evaluate local pulmonary function, a lung-perfusion single-photon-emission computed tomography (SPECT) and computed tomography (SPECT/CT) is a reliable tool [5,6]. We recently examined the forced expiratory volume in 1 second (FEV_1_) of each lobe after segmentectomy by using a lung-perfusion SPECT/CT. The results showed that the FEV_1 _of the preserved lobes after resection of 1, 2, and 3 segments were decreased, respectively, to 50%, 35%, and 17% of the preoperative value [7]. Especially, the resection of 2 segments in the right upper lobe, which has only 3 segments, can only preserve one segment. Therefore, for patients with small peripheral c-T1N0M0 NSCLCs involving multiple segments, we attempted the resection of only subsegments involved by tumor, i.e. combined subsegmentectomy (CSS), to preserve pulmonary function by avoiding the resection of multiple segments. For example, if the tumor involved the subsegment 2b and 3a of the right upper lobe (Figure 1), we performed the resection of S2b and S3a subsegments. This study examined the results of CSS in patients with peripheral c-T1N0M0 NSCLCs, with special reference to tumor size, location of tumor, and postoperative pulmonary function, which were compared with that after the resection of multiple segments.

## Methods

### Eligibility

The Ethics Committees of Kumamoto University Hospital approved the study protocol for sublobar resection in patients with c-T1N0M0 NSCLC. Informed consent was obtained from all patients after a comprehensive discussion of the risks and benefits of the proposed procedures [8,9].

### Indications for Segmentectomy and Subsegmentectomy

The criteria for segmentectomy was the followings: (1) peripheral c-T1N0M0 NSCLCs less than 3 cm diameter; (2) intraoperative frozen section of lymph nodes showed no metastasis; and (3) surgical margin of at least 2 cm from the tumor can be taken using CSS. The CSS or multiple segmentectomy was further indicated for tumors involving multiple segments, which were identified on serial sections of the axial, sagittal, and coronal views of multidetector CT images using Digital Imaging and Communications in Medicine data.

### Combined Segmentectomy Procedure

Segmentectomy including CSS was performed via open thoracotomy under one-lung ventilation as follows: (1) Pulmonary arteries and bronchi with tumor involvement were identified; (2) After the entire lung had been inflated, bronchi of the involved segment or subsegment were ligated and cut to clarify the boundary between the subsegments to be preserved versus resected, according to a previously reported technique [10]; (3) One lung ventilation was restarted, which made the lung tissues designated for preservation lose gas and collapse while retaining the segments subsegments designated for resection to be inflated, thereby allowing the border between the segments or subsegments of the resecting versus preserving lung tissue to be clarified; (4) The lung was cut using electrocautery between the inflated lung tissue to be resected and the deflated one to be preserved, thereby enabling the resection of targeted segments or subsegments; and (5) Cut plane of the lung was covered with a polyglycol acid sheet (Neoveil: Gunze Ltd., Kyoto, Japan) and fibrin glue to prevent postoperative air leakage.

### Patients

During April 2005 - March 2009, 248 patients with c-T1N0M0 NSCLC were treated with surgery. Of them, 198 patients (79%) were treated by segmentectomy. Of the 198 patients, CSS was conducted in 32 patients (16%). The other 166 patients were treated by the resection of single segment (single segmentectomy, n = 97) or the resection of multiple segments (multiple segmentectomy, n = 69).

### Pulmonary Function Tests

Vital capacity (VC), forced vital capacity (FVC), and FEV_1 _were measured before and more than 6 months after surgery with the patient in a seated position using a dry rolling-seal spirometer (CHESTAC-9800DN; Chest Inc. Tokyo, Japan) according to American Thoracic Society standards [11].

### SPECT/CT

SPECT/CT system was composed of a commercially available gantry-free SPECT with dual-head detectors (Skylight; ADAC Laboratories, Milpitas, Calif) and an 8-multidetector-row CT scanner (Light-Speed Ultra Instrument; General Electric, Milwaukee, Wis). Each 185 MBq of 99mTc-macroaggregated human serum albumin (Daiichi Radioisotope Laboratories, Ltd, Tokyo, Japan) was administered intravenously. The two scans were performed sequentially. The SPECT images were manually fused with the CT images on the workstation(AZE Virtual Place; AZE Co Ltd, Tokyo, Japan) [5,7].

Postoperative SPECT/CT was conducted with the pulmonary function test more than 6 months after surgery.

### Measurement of Pulmonary Function of Each Lobe

**Figure 2:**
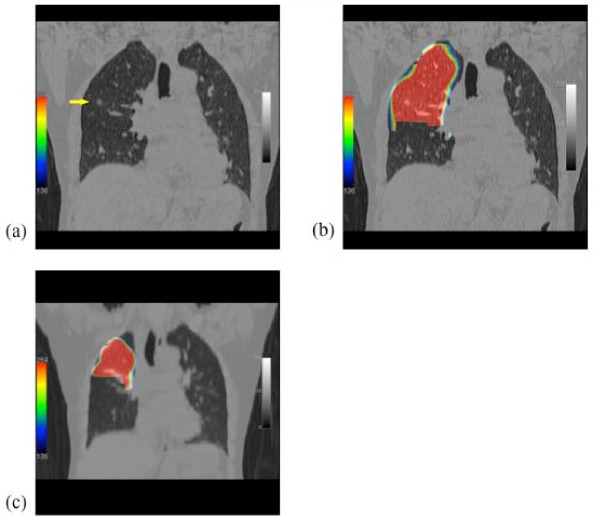
**Images of before and after segmentectomy**. (a) Coronal image of CT before surgery, showing a lung cancer (arrow) in the segment 2b of right upper lobe. (b) Coronal image of the perfusion SPECT/CT of the right upper lobe before operation. (c) Coronal image of the perfusion SPECT/CT of the remaining right upper lobe after the resection of S2b and S3a.

Images of the lobe before segmentectomy and of the lobe remaining after segmentectomy were traced on the CT image with a region of interest, of which radioisotope (RI) was counted on the SPECT image (Figure 2).

The FEV_1 _of the lobe before (A) and after (B) segmentectomy was measured from the preoperative or postoperative SPECT/CT according to the following formulae. A = Preoperative FEV_1 _× [RI counts of the lobe/RI counts of the whole lung]

B = Postoperative FEV_1 _× [RI counts of the lobe/RI counts of the whole lung]

The postoperative FEV_1 _of the lobe per preserved subsegment (C) was measured according to the following formula.

C = B/number of preserved subsegments of the lobe

### Statistical Analysis

Student's *t*-test was used to compare the tumor size, number of the resected or preserved subsegments, and preserved FEV_1 _among the CSS, single segmentectomy, and multiple segmentectomy. Pearson's χ^2 ^test was used to compare the location of tumors among the three groups. The SPSS software (SPSS Inc., Chicago, Illinois) was used for these analyses. Values of *p *< 0.05 were accepted as significant. All values in the text and table are given as mean ± SD.

## Results

Of the 32 patients who underwent the CSS, 17 patients who underwent both the pulmonary function test and lung-perfusion SPECT/CT both before and after surgery. Table 1 presents their resected sites and the number of resected subsegments. Mean number of the resected subsegments was 2.9 ± 1.1. If the entire segments involved by tumors were resected, the mean number of resected subsegments would be 5.0 ± 1.2, i.e. the CSS could save 2.2 ± 1.2 subsegments compared with the resection of entire segments.

**Table 1:** Sites of combined subsegmentectomy and the number of resected subsegments

	Resected sites	No. of resected SS	No. of patients
Right			
Upper lobe	S2b+S3a	2	3
	S2+S1a	3	2
	S1a+S2b+S3a	3	1
	S1b+S3a	2	1
	S1b+S3b	2	1
	S3+S2a	3	1
Lower lobe	S6b+S8a	2	1
	S6b+S8a+S9a	3	1
	S8a+S9a	2	1
			
Left			
Upper lobe	S1+2+S3a	4	1
	S1+2c+S3a	2	1
	S1+2c+S3b	2	1
	S3+S1+2a	4	1
Lower lobe	S9+S10+S8a	6	1

Total			17

SS: subsegment, CSS: combined subsegmentectomy

Of the 97 patients who underwent the single segmentectomy, 56 patients who underwent both the pulmonary function test and a lung-perfusion SPECT/CT both before and after surgery (Table 2). Of the 69 patients who underwent the multiple segmentectomy, 41 patients who underwent both the pulmonary function test and a lung-perfusion SPECT/CT both before and after surgery (Table 3).

**Table 2:** Sites of single segmentectomy and the number of resected subsegments

	Resected sites	No. of resected SS	No. of patients
Right			
Upper lobe	S1	2	2
	S2	2	4
	S3	2	9
Lower lobe	S6	3	10
	S8	2	4
	S9	2	2
	S10	3	1
			
Left			
Upper lobe	S1+2	3	10
	S3	3	6
	S4	2	1
Lower lobe	S6	3	6
	S8	2	1

Total			56

SS: subsegment

**Table 3:** Sites of multiple segmentectomy and the number of resected subsegments

	Resected sites	No. of resected SS	No. of patients
Right			
Upper lobe	S1+S2	4	3
	S2+S3	4	1
Lower lobe	S6+S8	5	1
	S6+S9	5	1
	S7+S8	4	1
	S9+S10	5	1
	S6+S9+S10	8	1
	S8+S9+S10	7	1
	S7+S8+S9+S10	9	2
			
Left			
Upper lobe	S1+2+S3	6	14
	S4+5	4	8
Lower lobe	S8+S9	4	4
	S9+S10	5	2
	S8+S9+S10	7	1

Total			41

SS: subsegment

Table 4 presents a comparison of preoperative pulmonary function, tumor size, location of tumor, and the numbers of resected and preserved subsegments among the CSS, single segmentectomy, and multiple segmentectomy. No significant difference of preoperative pulmonary function tests was found among these groups. Mean tumor size was 1.4 ± 0.5 cm in the CSS group, which was significantly smaller than the 2.0 ± 0.8 cm in multiple segmentectomy (p = 0.002). Location of tumor in the right upper lobe was 9 of the 17 (53%) patients who underwent the CSS, which was more frequent than 2 of the 41 (5%) who underwent the multiple segmentectomy (p < 0.001). Mean number of the resected subsegments in the CSS group was 2.9 ± 1.1, which was significantly less than 5.3 ± 1.4 of the multiple segmentectomy group (*p *< 0.001). However, the mean numbers of preserved subsegments of each lobe were not significantly different between the CSS and multiple segmentectomy (5.4 ± 2.5 vs. 5.0 ± 1.5). This discrepancy of the numbers of resected and preserved subsegments was caused by the difference of location of tumor, i.e. (1) Although the right upper lobe has only 6 subsegments, the right lower lobe, the left upper lobe, and the left lower lobe have 12, 10, and 10 subsegments, respectively, which makes the segmentectomy for the right upper lobe to preserve fewer subsegments than that for other lobes; and (2) Right upper lobe was the resected site more frequent in the CSS than in the multiple segmentectomy (53 vs. 5%, p < 0.001), causing the discrepancy of numbers of resected and preserved subsegments between the two groups.

**Table 4:** Patients' characteristics of combined subsegmentectomy, single segmentectomy, and multiple segmentectomy

	CSS	Single S	Multiple S
Mean age (y.o.)	61 ± 9	67 ± 11	71 ± 8
			
Sex			
Male	8	25	16
Female	9	31	25
			
Pulmonary function			
VC (L)	3.2 ± 0.7	3.0 ± 0.7	3.0 ± 0.9
%VC	111 ± 12	100 ± 13	112 ± 18
FEV_1 _(L)	2.4 ± 0.6	2.1 ± 0.5	2.0 ± 0.6
FVC/FEV_1_	75 ± 7	75 ± 7	71 ± 12
			
Mean tumor size (cm)	1.4 ± 0.5	1.7 ± 0.8	2.0 ± 0.8^†^
			
Location of tumor			
Right upper lobe	9	17	2^††^
Right lower lobe	3	15	10
Left upper lobe	4	17	22
Left lower lobe	1	7	7
			
Mean number of resected subsegments	2.9 ± 1.1	2.6 ± 0.6	5.3 ± 1.4^††^
			
Mean number of preserved subsegments	5.4 ± 2.5	6.8 ± 2.2	5.0 ± 1.5

Total	17	56	41

CSS: combined subsegmentectomy, Single S: single segmentectomy, Multiple S: multiple segmentectomy, VC: vital capacity, FVC: functional vital capacity, FEV_1_: forced expiratory volume in 1 second

†: p = 0.002 between the CSS and the multiple segmentectomy, ††: p < 0.001 between the CSS and the multiple segmentectomy.

**Figure 3:**
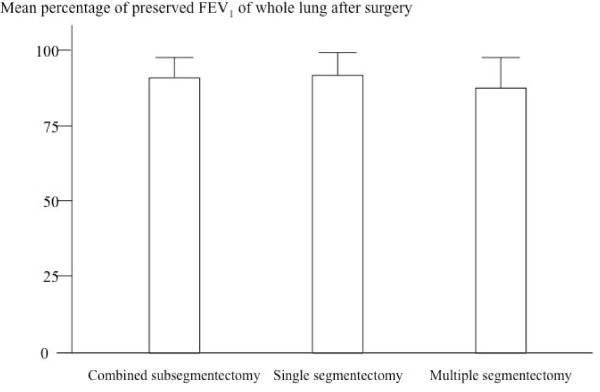
**Forced expiratory volume in 1 second examined by pulmonary function tests before and after surgery**.

Figure 3 shows the mean percentage of preserved FEV_1 _of whole lung after surgery in the three groups. In the CSS group, the mean values of FEV_1_of the whole lung before and after surgery were 2.4±0.6 and 2.2 ± 0.5 l, respectively, of which the mean percentage of FEV_1 _preserved, was 91 ± 7%. In the single segmentectomy group, the mean values of FEV_1 _of the whole lung before and after surgery were 2.2 ± 0.6 and 2.0 ± 0.5 l, respectively, of which the mean percentage of FEV_1 _preserved was 92 ± 8%. In the multiple segmentectomy group, the mean values of FEV_1 _of the whole lung before and after surgery were 2.0 ± 0.6 and 1.8 ± 0.6 l, respectively, of which the mean percentage of FEV_1 _preserved was 88 ± 10%. No significant difference of the mean percentage of FEV_1 _preserved was found among these three groups.

**Figure 4:**
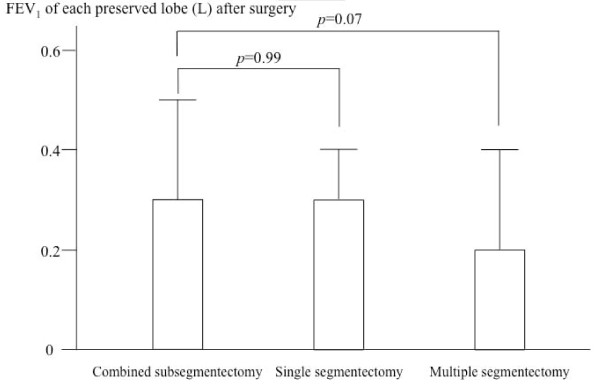
**Forced expiratory volume in 1 second of each lobe after surgery**.

Figure 4 shows the FEV_1 _of each preserved lobe after surgery in the three groups. In the CSS group, the mean values of FEV_1 _of each lobe before and after surgery were 0.6 ± 0.2 and 0.3 ± 0.2 l, respectively. In the single segmentectomy group, the mean values of FEV_1 _of each lobe before and after surgery were 0.5 ± 0.2 and 0.3 ± 0.1 l, respectively. In the multiple segmentectomy group, the mean values of FEV_1 _of each lobe before and after surgery were 0.5 ± 0.2 and 0.2 ± 0.2 l, respectively. While there was no significant difference of the postoperative FEV_1 _of each lobe between the CSS and single segmentectomy, the value of the CSS was higher than that of the multiple segmentectomy with marginal significance (p = 0.07).

**Figure 5:**
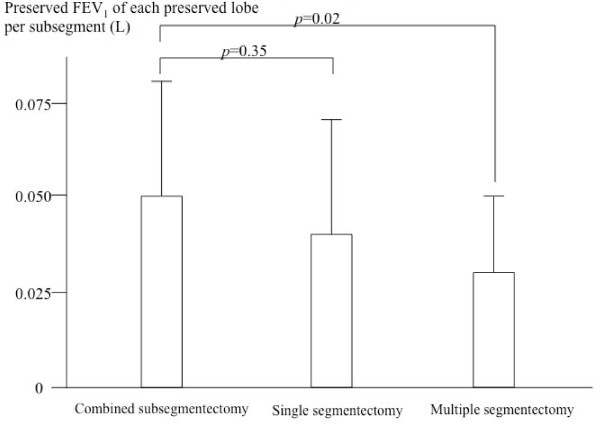
**Preserved forced expiratory volume in 1 second of each lobe per subsegment after surgery**.

Figure 5 shows the FEV_1 _of each preserved lobe per subsegment after surgery, which were 0.05 ± 0.03, 0.04 ± 0.03, 0.04 ± 0.03 l in the CSS, single segmentectomy, and multiple segmentectomy, respectively. The value was significantly higher in the CSS than in the multiple segmentectomy (p = 0.02).

All of the 198 patients who underwent CSS, single segmentectomy, or multiple segmentectomy were discharged from the hospital without major complications. All of the tumors were pathologically N0 stage. With the mean follow-up period after surgery was 31 ± 10 months (range: 12-60 month), 5 of the 166 patients (2%) who underwent single or multiple segmentectomy suffered postoperative recurrence, but there was no recurrence at the surgical margin. All 32 patients who underwent CSS are alive without recurrence.

## Discussion

Results of this study elucidated the following points: (1) The CSS could save 2.2 ± 1.2 subsegments compared with the resection of entire segments involved by tumors; and (2) Both the preserved FEV_1 _of each lobe and that value per subsegment were higher in the CSS than in the multiple segmentectomy, whereas there was no significant difference of preserved % of FEV_1 _of whole lung between the two groups.

The reason for no significant difference in pulmonary function between the CSS and multiple segmentectomy could be caused by the difference of frequency of right upper lobe between the two. The CSS was conducted for right upper lobe more frequently than the multiple segmentectomy, because the right upper lobe has fewer subsegments than the other lobes. To preserve sufficient lung tissue for tumors involving multiple segments in the right upper lobe, we conducted the CSS frequently, for example the resection of S2b and S3a rather than the resection of both the S2 and S3. Contrary to the right upper lobe, other lobes can preserve sufficient lung tissue even after multiple segmentectomy, because they have more subsegments than the right upper lobe. Therefore, our data show that the CSS could preserve the pulmonary function of each lobe by avoiding the multiple segmentectomy, especially for tumors in the right upper lobe.

The mean values of postoperative FEV_1 _after CSS, single segmentectomy, and multiple segmentectomy were approximately 90% of the preoperative values, which were comparable to values in the previous reports of general segmentectomy [7,12,13] and were higher than that after lobectomy [12]. We previously reported that the postoperative FEV_1 _of each lobe after the resection of 1, 2, and 3 segments was decreased to 50%, 35%, and 17%, respectively [7]. The use of CSS can obviate the resection of multiple segments that a tumor involves. Therefore, to preserve a pulmonary function after segmentectomy in patients with small peripheral c-T1N0M0 NSCLC involving multiple segments, CSS would be preferable to the resection of multiple segments with tumor involvement, especially for small tumors located in the right upper lobe.

This study revealed that the mean tumor size in the CSS was significantly smaller than that in multiple segmentectomy. The mean tumor size in the CSS group was 1.4 ± 0.5 cm, ranging from 0.8 to 2.4 cm. To take the surgical margin of at least 2 cm from the tumor by the CSS, tumors larger than 2 cm involving multiple segments would be out of the indication for CSS.

The disadvantage of the CSS might be that the lymph node dissection at hilum of resected subsegments would be less sufficient than the conventional segmentectomy. Therefore, we recommend it for likely pathological N0 tumors, such as bronchioloalveolar carcinoma, carcinoid, and metastatic pulmonary tumors.

The preserved pulmonary functions after CSS, single segmentectomy, and multiple segmentectomy are shown herein. Our data indicate that the CSS is useful for preservation of pulmonary function of each lobe by avoiding the multiple segmentectomy especially in patients with small sized tumors with likely pathological N0 involving multiple segments of the right upper lobe.

## Abbreviations

NSCLCs: non-small cell lung cancers; CSS: combined subsegmentectomy; FEV_1_: forced expiratory volume in 1 second; SPECT/CT: lung-perfusion single-photon-emission computed tomography and computed tomography; RI: radioisotope.

## Competing interests

The authors declare that they have no competing interests.

## Authors' contributions

This report reflects the opinion of the authors and does not represent the official position of any institution or sponsor. The contributions of each of the authors were as follows: KY was responsible for reviewing previous research, journal handsearching, drafting report. HN was responsible for quality checking and data processing. HN was responsible for project coordination. All authors have read and approved the final manuscript.
